# The pattern of injury and poisoning in South East Iran

**DOI:** 10.1186/1472-698X-12-17

**Published:** 2012-09-10

**Authors:** Alireza Ansari-Moghaddam, Alexandra LC Martiniuk, Mahdi Mohammadi, Mahdieh Rad, Fatemeh Sargazi, Khodadad Sheykhzadeh, Seddighe Jelodarzadeh, Fatemeh Karimzadeh

**Affiliations:** 1 Health Promotion Research Center, Zahedan University of Medical Sciences, Zahedan, Iran; 2 The George Institute for Global Health at the University of Sydney, M201 Missenden Rd, Camperdown, NSW, 2050, Australia; 3 Dalla Lana School of Public Health, University of Toronto; Sunnybrook Health Sciences Centre, Toronto, Canada

**Keywords:** Iran, Injury, Road traffic crash, Urban/rural, Sex

## Abstract

**Background:**

Injury is a leading cause of morbidity and mortality worldwide, and even more so in low and middle-income countries (LMICs). Iran is a LMIC and lacks information regarding injury for program and policy purposes. This study aimed to describe the incidence and patterns of injury in one province in South Eastern Iran.

**Methods:**

A hospital-based, retrospective case review using a routinely collected registry in all Emergency Departments in Sistan and Baluchistan province, Iran for 12 months in 2007–2008.

**Results:**

In total 18,155 injuries were recorded during the study period. The majority of injuries in South Eastern Iran were due to road traffic crashes. Individuals living in urban areas sustained more injuries compared to individuals from rural areas. Males typically experienced more injuries than females. Males were most likely to be injured in a street/alley or village whereas females were most likely to be injured in or around the home. In urban areas, road traffic related injuries were observed to affect older age groups more than younger age groups. Poisoning was most common in the youngest age group, 0 to 4 years.

**Conclusions:**

This study provides data on incidence and patterns of injury in South Eastern Iran. Knowledge of injury burden, such as this paper, is likely to help policy makers and planners with health service planning and injury prevention.

## Background

Injuries are a large and increasing health problem worldwide, causing more than 5 million deaths annually; 16,000 deaths every day [1,2]. Furthermore, it has been estimated that deaths from injuries will increase from 5.1 million to 8.4 million (9.2% of all global deaths), with road traffic injury alone being the third leading cause of death and disability adjusted life years (DALYs) by the year 2020 [3-5]. Notably, the greatest burden of injuries is concentrated in low- and middle income countries where the least evidence and resources are available for preventive measures and care following injury [2]. More importantly, current estimations show that the pattern of injury morbidity and mortality due to road traffic crashes are decreasing by about 27% in high income countries, but increasing by more than 80% in low and middle income countries [6,7].

Several epidemiological studies examining the incidence and pattern of injuries have suggested that the magnitude, characteristics and pattern of injury vary considerably from country to country [5,8-17]. Yet, injury as a research problem has been largely ignored in developing countries such as Iran. Recent research has found the annual road traffic death rate to be 44 per 100,000 in Iran, higher than any other death rate in any region of the world where reliable estimates of road traffic crashes are available [18]. While this study, and also others, such as the global burden of injuries [2] provide estimates for injury mortality in Iran, few provide data on non-fatal injuries, types of injury, by sex and by urban or rural residence. Therefore, this study aimed to provide data on the incidence and patterns of injury in the Sistan and Baluchistan Province of Iran. This province is a low income region in the South East of Iran [19].

## Methods

A registry-based, retrospective study was used, reviewing all individuals admitted for injury between 20 March 2007 to 19 March 2008 (One Iranian Year) to all emergency departments of the Ministry of Health and Medical Education of Iran in Sistan and Baluchestan province. As such this was a hospital-based study for the full population of the province. The province is located in South-East region of Iran at the border Afghanistan and Pakistan from the West and Oman Sea from the South [18]. The population in the Sistan and Baluchistan province during the study period was 2.1 million. (Excluding the population of Zabol which has been recently disintegrated from the province) [19]. Overall, there were 12 emergency rooms in 7 districts of the province where injured patients admitted during the study period. Patients were included in this study if they initially arrived, alive, into the emergency department. Their injuries are included in this study, even if they died later, as a result of their injuries. Individuals who died prior to arrival at the emergency department were not included in this study.

In each emergency room, trained staff complete the nationally designed medical forms for each injured patient admitted to hospital. Injury is consistently defined across all hospitals in the province, as injury to any body part leading an individual to seek medical care in the Emergency Department.

The data register thus contains data on demographic details as well as type, cause of injury, place of injury, place of residence (urban/rural) and the outcome of injury. Injuries were classified according to the 10^th^ revision (ICD-10-E) of the International Classification of Diseases. Patient data were extracted, using unique identifiers to protect patient confidentiality, by two research assistants under the supervision of the lead investigator. Collected data were transferred to the Center for Disease Control and prevention of health centers in each district. In each provincial health center, one expert (i.e. bachelor of public health) was responsible for data gathering and quality control. Trauma was defined in this study as any physical damage/injury/wound to the body caused unintentionally by contact with an object (i.e. struck accidentally by object or bumping into or against object) excluding injury caused by assault and transport vehicle. Those counted as being injured in a road traffic crash included pedestrians, motorcyclists and vehicle occupants.

Data were stored and analyzed using SPSS Version 15. Descriptive statistics were examined to explore the frequency distribution of data. Age standardized incidence rates and 95% confidence intervals (CI) of injuries were calculated. Incidence rates were standardized using the direct standardization method [20] for event rates and using the whole population of Iran [21] as a standard population.

This study was approved by the research ethics committee of Zahedan University of Medical Sciences, Iran. Written informed consent was obtained from the patient for publication of this report and any accompanying images. Study participants were each given a unique identifier and personal data were only accessible to the principal investigators.

## Results

Table 1 presents the age standardized incidence rates for types of injury by sex and place of residence. In total 18,155 injuries were recorded during the study period. The overall age-standardized incidence rate for all injuries was 916 (95% CI 902–930) per 100,000. The majority of these injuries were due to road traffic crashes 5,713 (32%). The next most common injuries were trauma 3,605 (20%) and poisoning 2,390 (13%). For all injury types, people living in urban areas were more likely to present to the emergency department for injury compared to individuals living in rural areas. For most injury types, males were more likely than females to be affected. However, the male to female ratio was closer to 1:1 for poisoning, injuries involving animals and burns.

**Table 1:** Age standardized incidence rate (ASIR) of injuries by sex and place of residence

**ASIR Type of injury**	**Total**		**Place of residence**			**Sex**		
			**Urban**	**Rural**	**Rate ratio Urban: Rural**	**Male**	**Female**	**Rate ratio Males: Females**
	**n (%)**	**Rate (95% CI)**	**Rate (95% CI)**	**Rate (95% CI)**		**Rate (95% CI)**	**Rate (95% CI)**	
All injuries	18155 (100)	916 (902–930)	1375 (1351–1399)	250 (239–261)	5.5 : 1	1360 (1336–1384)	468 (453–482)	2.9: 1
Traffic	5713 (31.5)	308 (299–316)	390 (377–403)	87.5 (81.0-94.0)	4.5 : 1	514 (499–529)	99.3 (92.4-106)	5.2: 1
Trauma	3605 (19.9)	176 (170–182)	307 (295–318)	24.5 (21.1 - 27.9)	12.5: 1	274 (263–284)	76.7 (70.8 - 82.7)	3.6: 1
Poisoning	2390 (13.2)	113 (108–117)	191 (182–199)	22.5 (19.4 - 25.6)	8.5 : 1	127 (120–134)	98.8 (92.5 - 105)	1.3 : 1
Falls	1676 (9.20)	78.7 (74.7 - 82.8)	121 (114–128)	32.3 (28.4 - 36.2)	3.7 : 1	118 (112–125)	38.1 (33.8 - 42.3)	3.1 : 1
Animal involved	1135 (6.25)	57.9 (54.3 - 61.5)	75.0 (69.3 - 80.6)	36.2 (32.1 - 40.4)	2.1 : 1	54.1 (49.4 - 58.9)	62.2 (56.8 - 68.8)	0.87 : 1
Violence/assault	1113 (6.10)	60.3 (56.6 - 63.9)	95.3 (89.1 - 102)	15.9 (13.2 - 18.7)	6.0 : 1	109 (102–116)	11.5 (9.18 - 13.8)	9.5 : 1
Burns	596 (3.25)	27.6 (25.2 - 29.9)	44.5 (40.3 - 48.8)	7.60 (5.70 - 9.40)	5.9 : 1	31.3 (27.8 - 34.9)	23.8(20.6 - 27.0)	1.3 : 1
Others	1927 (10.6)	95.6 (91.1 - 100)	152 (144–160)	23.3 (20.0 - 26.5)	6.5 :1	133 (126–141)	57.3 (52.2 - 62.4)	2.3 :1

Note: Rates are per 100,000 persons; CI, confidence interval; others including suicide, drowning, Exposure to electricity etc. Animal involved, bitten, stung or attacked by an animal.

Table 2 presents the age standardized incidence rate for injury by place, sex and location of residence. Most injuries occurred in or near the home 7,877 (43%). Thus, the highest age-standardized incidence rate was observed for injuries occurring in or near the home 371 (95% CI 362–380) per 100,000. Males were most likely to be injured in a street/alley or village, rate 509 (95% CI 495–524) whereas females were most likely to be injured in or around the home, rate 320 (95% CI 308–332). Urban rates were consistently higher than rural age-standardized injury rates for all injury locations examined.

**Table 2:** Age standardized incidence rate (ASIR) of Injury by place, sex and residency

**ASIR Place of injury**	**Total**		**Place of residence**		**Sex**	
			**Urban**	**Rural**	**Male**	**Female**
	**n (%)**	**Rate (95% CI)**	**Rate (95% CI)**	**Rate (95% CI)**	**Rate (95% CI)**	**Rate (95% CI)**
Home (Inside/outside)	7877 (43.4)	371 (362–380)	634 (618–650)	90.4 (84.0 - 96.8)	421 (409–434)	320 (308–332)
Street/alley inside city or village	5673 (31.2)	297 (289–305)	495 (480–509)	75.2 (69.2 - 81.2)	509 (495–524)	81.6 (74.7 - 86.7)
On the road outside city or village	1947 (10.7)	111 (106–116)	53.3 (48.4 - 58.1)	32.5 (28.5 - 36.5)	186 (177–195)	34.3 (30.2 - 38.4)
Workplace/factory	743 (4.10)	41.5 (38.5 - 44.6)	66.4 (61.1 - 71.7)	8.50 (6.34 - 10.6)	81.0 (74.7 - 86.7)	2.10 (1.13 -3.06)
School	293 (1.60)	12.3 (10.8 - 13.7)	18.5 (16.0 - 20.9)	5.27 (3.93 - 6.60)	19.8 (17.2 - 22.4)	4.58 (3.33- 5.83)
Recreation area including sports	199 (1.10)	9.60 (8.20 - 10.9)	14.7 (12.3 - 17.0)	3.32 (2.10 - 4.55)	17.2 (14.6 - 19.8)	1.84 (0.95 - 2.74)
Others	1423 (7.90)	74.5 (70.5 - 78.5)	93.9 (87.6 - 100)	34.5 (30.5 - 38.6)	125 (118–133)	23.0 (19.8 - 26.1)
Total	18155 (100)	916 (902–930)	1375 (1351–1399)	250 (239–261)	1360 (1336–1384)	468 (453–482)

Note: Rates are per 100,000 persons; CI, confidence interval.

**Figure 1:**
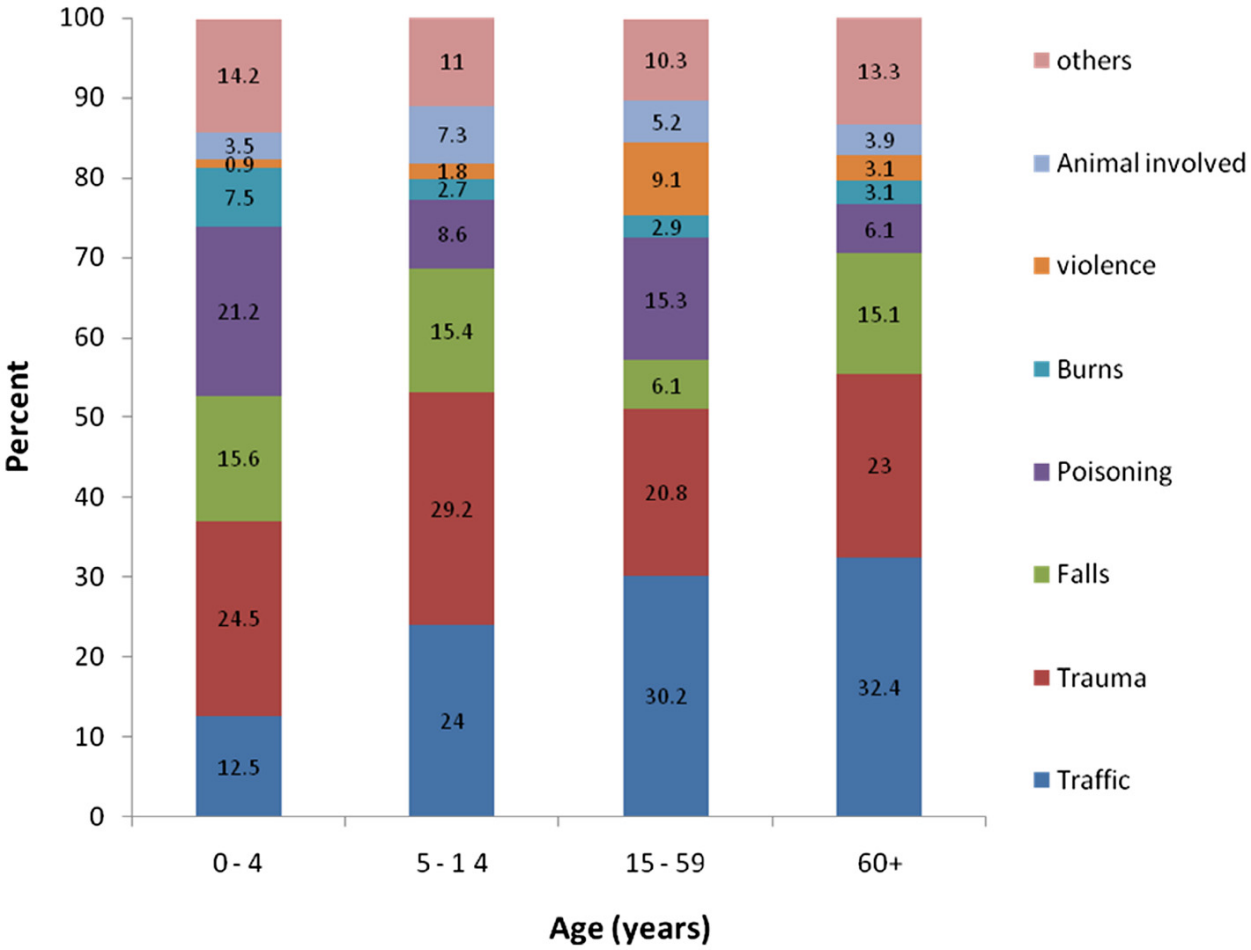
(Urban).

**Figure 2:**
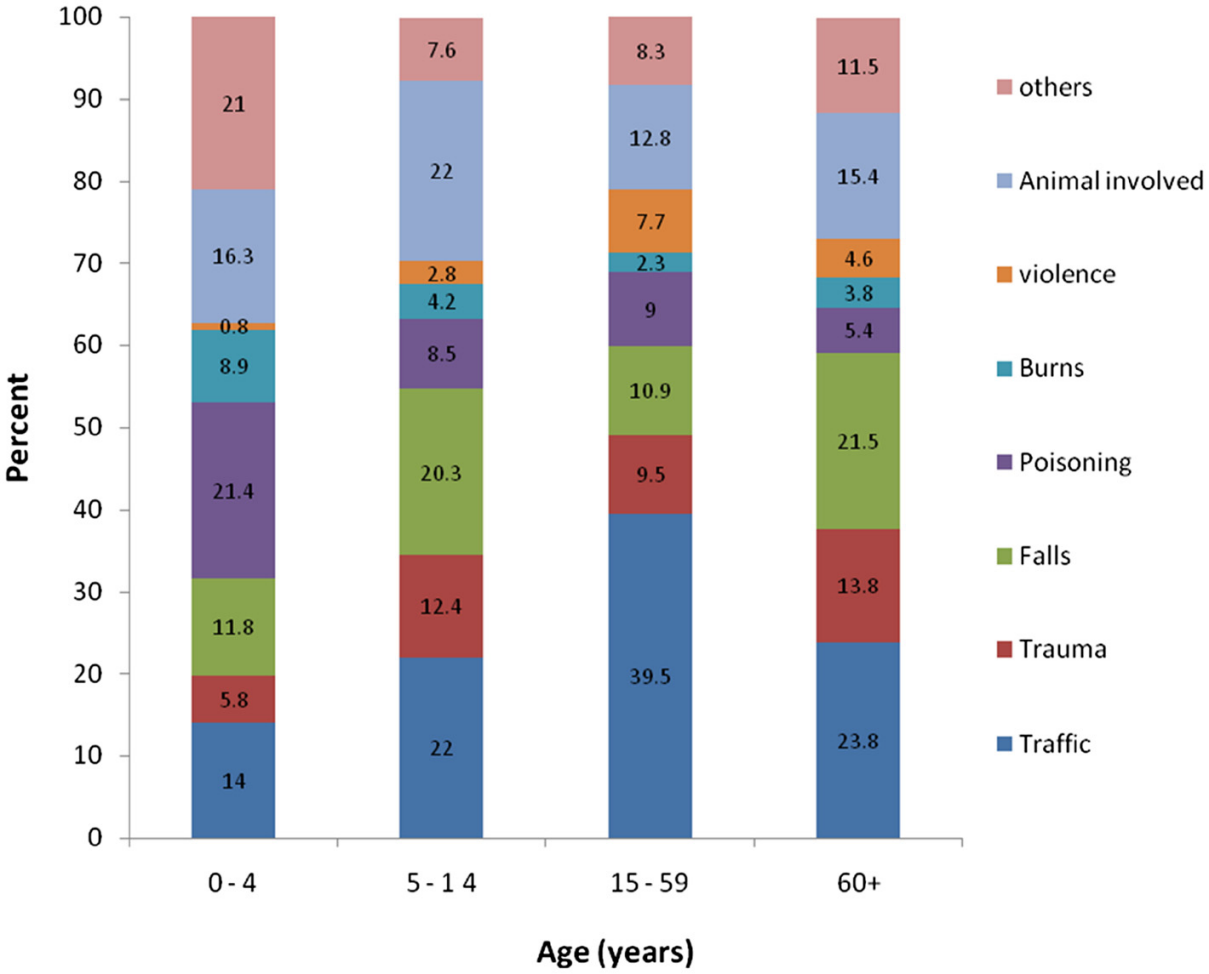
(Rural).

Figure 1 presents the percent of injuries occurring by age and by type of injury for individuals living in urban locations. In urban areas, road traffic related injuries were observed to affect older age groups more than younger age groups. Poisoning was most common in the youngest age group, 0 to 4 years. Figure 2 presents the percent of injuries occurring by age and by type of injury for individuals living in rural locations. The rural distribution of injury mechanism by age group differed from urban areas. For instance, road traffic related injuries were more common in the 15–59 year age group in rural areas (compared to the oldest age group for road traffic injuries in urban areas). The present study observed that traffic related injuries were more likely to result in death compared to injuries due to other external causes. Overall, a total of 106 (0.6%) injury deaths occurred among the surveyed population. Road traffic injuries were responsible for approximately half of the all injury deaths (49/106).

## Discussion

This study found that the majority of injuries in South Eastern Iran were due to road traffic crashes. The next most common injuries were trauma and poisoning. For all injury types, individuals living in urban areas were more likely to be injured compared to rural areas. For most injury types, males were more likely than females to be affected. Males were most likely to be injured in a street, alley or village whereas females were most likely to be injured in or around the home. In urban areas, road traffic related injuries were observed to affect older age groups more than younger age groups. Poisoning was most common in the youngest age group, 0 to 4 years.

The pattern of injuries observed in our study, with road traffic crashes, trauma and poisoning being the most common, differ from fires, drowning, violence and war-related injuries which are the most common injuries in Sub-Saharan Africa; and self-inflicted injuries and traffic-related injuries which are the most common in China and Latin America [8]. There are mixed results about the occurrence of injury by urban/rural place of residence in the literature [5,11,12]. Similar to our findings, the vast majority of previously published studies also show that males are at a higher risk of having an injury than females [5,8-17,22]. Previous studies have also shown that most injuries occur in and around home [5,8-17,22].

Our study found the overall injury rate to be 916 per 100,000 individuals. This rate is similar to the injury rate observed in a recent study of nearly 3 million emergency department admissions for injury in Iran; approximately 1% per year in our study and 1.4% in the study by Rasouli et al. [23]. However, the rate we observed for injury is much higher than the non-fatal injury rate reported previously in a national study in Iran of 444.3 per 100,000 [24]. A smaller study in a rural state of Iran found the non-fatal injury rate to be 172 per 100,000 people [16]. However, these non-fatal injury rates observed in Iran are still much lower than the 10,000 per 100,000 individuals reported in a study in rural Nigeria [14]. The difference in observed rates in our study compared to the previous Iranian study could be due to the studied province truly having increased rates of injury or could also be due to the type of data used to calculate injury rates. The previous study by Soori et al. [24] used a retrospective survey of households across Iran, whereas our study used emergency room medical records. Although we would expect a household survey to capture more, rather than less injuries, given household surveys are more likely to include less severe injuries as well as the more severe injuries presenting to emergency departments. The sex differences observed in our study are in keeping with past research internationally as well as in Iran, which demonstrate that males have increased risk of injury compared to females [24]. Our study observed an increased injury rate for individuals living in urban, compared to rural, areas at a ratio of about 5 to 1. In comparison, the national Iranian study found injury rates to be approximately equal across urban and rural areas [24].

Our findings regarding road traffic crashes being the most common risk factor for injury mirrors that previously observed in Iran [16,18] and around the world [2,14,25]. We observed a road traffic injury rate of 308 per 100,000. A previous study found that in Iran, injuries on the road were due mainly to motorized two-wheeler riders, whereas deaths were mainly due to cars [18]. In a previous study in Pakistan a road traffic injury rate of 1,500 per 100,000 was observed [11]. The study in Pakistan was a nationally representative survey study of households. Similar to our study, the study of injuries in Pakistan also found a male predominance in road traffic crashes and also found young and older adults to be at higher risk (compared to children). A recently published article investigating factors related to road traffic injury severity in Iran found that lack of seat belt use was the most important factor related to injury severity [26]. Improper overtaking, speeding, vehicle defects and unauthorized vehicles on freeways, as well as pedestrians and livestock were also factors which increased injury severity [26].

### Strengths and limitations

This study provides information from a large dataset comprising over 18,000 injuries from South Eastern Iran. By collecting data at all emergency departments in the province, we are able to provide incidence data for injuries (severe enough to lead to emergency room presentation) and to examine patterns of injury by age, sex and urban/rural status. Data were routinely collected from medical records therefore were standardized across different emergency rooms. However, since this study collected data from emergency departments it was not able to capture less severe injuries, or those treated traditionally in the community. Furthermore, it was not possible to determine the outcome of patients in details due to insufficient data in this case. Data were coded according to the ICD-10 which facilitates comparisons with other regions and countries.

## Conclusion

This study provides rigorous data on incidence and patterns of injury in South Eastern Iran. Data on non-fatal injuries in Iran, and most lower-and middle-income countries is lacking and thus information from this paper assists in filling local and international knowledge gaps. Information on the size and type of the injury burden in Iran will also help policy makers and planners with health service planning and injury prevention. Future research could include other regions in Iran as well as provision of ICD external cause codes. As always, for policy makers, future research to estimate the economic costs of injuries in Iran will be useful for prioritization of prevention and care.

## Competing interests

The authors declare that they have no competing interests.

## Authors’ contributions

KS and FS supervised data collection. SJ, FK, and MR conducted the literature review and data entry. MM, AAM and MR analysed and interpreted data. AAM, AM drafted the first version of manuscript. All authors read and approved the final manuscript.

## Contributorship

This work was supported by the staff in all emergency departments in the Sistan and Baluchistan province who collected the data. AAM, MM, MR, FS, KS, SJ, FK were supported by their home institution: Zahedan University of Medical sciences, Zahedan, Iran. AM receives salary support in the form of an unrestricted educational fellowship from Merck.

## Appendix

What this study adds (BOX)

What is already known on this subject

Injury is a leading cause of morbidity and mortality worldwide, and even more so in developing countries

Several studies exist in Iran which examine fatal injuries but less is known about non-fatal injuries

What this study adds

Examining over 18,000 injuries occurring in 2007–2008 in south eastern Iran, majority of injuries were due to road traffic crashes, followed by trauma and poisoning

Individuals living in urban areas were more likely to be injured compared to rural areas; and males more likely to be injured than females

In urban areas, road traffic related injuries were observed to affect older age groups more than younger age groups

## Pre-publication history

The pre-publication history for this paper can be accessed here:

http://www.biomedcentral.com/1472-698X/12/17/prepub

## References

[B1] World Health Organization Violence, Injuries, and Disability: Biennial 2006–2007 Report 2008 World Health Organization, Geneva

[B2] Chandran A Peek-Asa C Hyder AA The global burden of unintentional injuries and an agenda for progress Epidemiol Rev 2010 32 110 120 10.1093/epirev/mxq009 20570956 PMC2912603

[B3] World Health Organization Health statistics and health information systems – Projections of mortality and burden of disease to 2030 WHO, Geneva http://www.who.int/healthinfo/statistics/bodprojections2030/en/index.html

[B4] Murray CJ Lopez AD Alternative projections of mortality and disability by cause 1990–2020: global burden of disease study Lancet 1997 349 1498 1504 10.1016/S0140-6736(96)07492-2 9167458

[B5] Fatmi Z Hadden WC Razzak JA Qureshi HI Hyder AA Pappas G Incidence, patterns and severity of reported unintentional injuries in Pakistan for persons five years and older: results of the National Health Survey of Pakistan 1990–94 BMC Public Health 2007 7 152 10.1186/1471-2458-7-152 17623066 PMC1933417

[B6] Peden M Global collaboration on road traffic injury prevention Int J Inj Contr Saf Promot 2005 12 85 91 10.1080/15660970500086130 16156532

[B7] Peden M McGee K Sharma G The injury chart book: a graphical overview of the global burden of injuries 2002 World Health Organization, Geneva

[B8] Hang HM Epidemiology of unintentional injuries in Rural Vietnam. Department of Public Health and Clinical Medicine 2004 Umea University, Sweden

[B9] Mock CN Abantanga F Cummings P Koepsell TD Incidence and outcome of injury in Ghana: a community-based survey Bull World Health Organ 1999 77 955 964 10680242 PMC2557773

[B10] Hang HM Bach TT Byass P Unintentional injuries over a 1-year period in a rural Vietnamese community: describing an iceberg Public Health 2005 119 466 473 10.1016/j.puhe.2004.08.022 15826887

[B11] Ghaffar A Hyder AA Masud TI The burden of road traffic injuries in developing countries: the first national survey of Pakistan Public Health 2004 118 211 217 10.1016/j.puhe.2003.05.003 15003410

[B12] Moshiro C Ivar H Anne N Philip S Yusuf H Gunnar K Injury morbidity in an urban and a rural area in Tanzania: an epidemiological survey BMC Public Health 2005 5 11 10.1186/1471-2458-5-11 15679887 PMC548509

[B13] Kobusingye O Guwatudde D Lett R Injury patterns in rural and urban Uganda Inj Prev 2001 7 46 50 10.1136/ip.7.1.46 11289535 PMC1730690

[B14] Olawale OA Owoaje ET Incidence and pattern of injuries among residents of a rural area in South-Western Nigeria: a community-based study BMC Public Health 2007 7 246 10.1186/1471-2458-7-246 17875213 PMC2169237

[B15] Mohammadi R Ekman R Svanström L Gooya MM Unintentional home-related injuries in the Islamic Republic of Iran: findings from the first year of a national programme Public Health 2005 119 919 924 10.1016/j.puhe.2005.01.012 15939446

[B16] Shahkolai FR Naghavi M Shokouhi M Laflamme L Unintentional injuries in the rural population of Twiserkan, Iran: a cross-sectional study on their incidence, characteristics and Preventability BMC Public Health 2008 8 269 10.1186/1471-2458-8-269 18671856 PMC2533326

[B17] Rahimi-Movaghar V Controlled evaluation of injury in an international safe community: Kashmar, Iran Public Health 2010 124 190 197 10.1016/j.puhe.2010.02.014 20417350

[B18] Bhalla K Naghavi M Shahraz S Bartels D Murray CJL Building national estimates of the burden of road traffic injuries in developing countries from all available data sources: Iran Inj Prev 2009 15 150 156 10.1136/ip.2008.020826 19494093

[B19] Wikipedia. Sistan and Baluchestan Province http://en.wikipedia.org/wiki/Sistan_and_Baluchestan_Province

[B20] Woodward M Epidemiology: Study design and data analysis 2004 2 Chapman and Hall/CRC, Boca Raton

[B21] Iranian statistical center. General census on the population and houses, 2006 http://www.amar.org.ir/nofous1385/default-2458.aspx

[B22] Taghavi M Rasouli MR Boddouhi N Zarei MR Khaji A Abdollahi M Epidemiology of outpatient burns in Tehran: an analysis of 4813 cases Burns 2010 36 109 113 10.1016/j.burns.2009.02.011 19818561

[B23] Rasouli MR Saadat S Haddadi M Epidemiology of injuries and poisonings in emergency departments in Iran Public Health 2011 125 727 733 10.1016/j.puhe.2011.07.006 21906762

[B24] Soori H Akbari ME Ainy E Zali AR Naghavi M Shiva N Epidemiological pattern of non-fatal injuries in Iran Pak J Med Sci 2010 26 206 211

[B25] Krug EG Sharma GK Lozano R The global burden of injuries Am J Public Health 2000 90 523 526 10754963 10.2105/ajph.90.4.523 PMC1446200

[B26] Kashani AT Shariat-Mohaymany A Ranjbari A Analysis of factors associated with traffic injury severity on rural roads in Iran J Inj Violence Res 2012 4 1 36 41 Epub 2011 Apr 16 21502788 10.5249/jivr.v4i1.67 PMC3291279

